# Prediction, Discovery, and Characterization of Plant- and Food-Derived Health-Beneficial Bioactive Peptides

**DOI:** 10.3390/nu14224810

**Published:** 2022-11-14

**Authors:** Martin Kussmann

**Affiliations:** 1 Kussmann Biotech GmbH, 59394 Nordkirchen, Germany; martin@kussmann.ch; 2 German Entrepreneurship, Cambridge, MA 02142, USA

**Keywords:** bioactive, peptide, nutrition, food, plant, ingredient, supplement, artificial intelligence, design, peptidomics, mass spectrometry, discovery, validation, in vitro biology, manufacturing, sustainability

## Abstract

Nature may have the answer to many of our questions about human, animal, and environmental health. Natural bioactives, especially when harvested from sustainable plant and food sources, provide a plethora of molecular solutions to nutritionally actionable, chronic conditions. The spectrum of these conditions, such as metabolic, immune, and gastrointestinal disorders, has changed with prolonged human life span, which should be matched with an appropriately extended health span, which would in turn favour more sustainable health care: “adding years to life and adding life to years”. To date, bioactive peptides have been undervalued and underexploited as food ingredients and drugs. The future of translational science on bioactive peptides—and natural bioactives in general—is being built on (a) systems-level rather than reductionist strategies for understanding their interdependent, and at times synergistic, functions; and (b) the leverage of artificial intelligence for prediction and discovery, thereby significantly reducing the time from idea and concept to finished solutions for consumers and patients. This new strategy follows the path from benefit definition via design to prediction and, eventually, validation and production.

## 1. Natural Bioactives from Plants and Foods

Natural bioactives can be classified into micronutrients (i.e., vitamins and minerals) [1]; phytonutrients (e.g., phenolics, alkaloids, and terpenes) [2,3]; pre- and probiotics [4]; and bioactive peptides [5]. In particular, bioactive peptides have remained largely underappreciated as molecular deliverers of health promotion, mainly due to [6]: (assumedly) poor bioavailability after oral consumption due to proteolysis along the gastrointestinal tract; limited transport from the gut lumen to the bloodstream; and, importantly, insufficient discovery and translation based on serendipitous research and/or high-throughput screening [6].

Natural bioactive plant and food peptides can be regarded as safe and efficacious means for the molecular delivery of specific health benefits, and they are well-suited for in silico prediction and discovery because [7]:

- Peptides can be regarded as the “vocabulary of nature”: living systems use peptides to communicate and to regulate and fine-tune their functions. Peptides have co-evolved with humans as modulators of physiology and therefore exert highly specific biological functions. The presence in natural (e.g., plant and food) sources and the biological function of peptides can be predicted in silico by blasting peptide sequences against the plant and food genomes and by the computational and human interpretation of metabolic and signalling pathways. Orally administered peptides often suffer from a short half-life across ingestion and digestion, as well as in the blood circulation. The challenges of using peptides as orally delivered bioactives lie in their stability, bioavailability, and bioefficacy, rather than in their safety. From a food perspective, peptides can be considered as nutrients, and they are the only nutrients that are directly encoded in the genomes of their sources [8]. Food peptides are components of long-term consumed food sources. Food protein hydrolysates can therefore be “generally recognized as safe” (GRAS) [8].

While peptides are present and consumed abundantly in foods, they have typically been discovered ad hoc or through traditional use. Presently, the most prominent applications of peptides in food are in infant formulae, sports nutrition, and dietary supplements [9]. Food protein hydrolysates containing bioactive peptides also have a long history of global use, e.g., in infant formulae based on soy or rice protein hydrolysates to avoid cow’s milk allergy; or fermented foods such as yogurt, kefir, kimchi, tempeh, tofu, natto, and pickled vegetables. The safety of food protein hydrolysates has been assessed in pre-clinical and clinical studies and they are regulated as food hydrolysates, not as drugs [8].

Fermented dairy products provide health benefits due to their peptide content: for example, peptides (in the form of hydrolyzed vegetable proteins) provide savoury flavours for traditional and processed foods; other anti-microbial peptides produced by the cheese-making process protect the food from spoilage. Furthermore, food-derived peptides are increasingly being accepted not only as providers of additional health benefits beyond macronutrient-based good nutrition, but also as a potential solution to the removal and/or substitution of artificial preservatives [10], sugar and salt reduction, and the enabling of cultured meat protein [8].

## 2. Artificial Intelligence in (Life) Science and Technology

Human intelligence is unmatched when it comes to versatility: the human brain is extremely flexible in learning and in developing and performing a vast array of cognitive, creative, and executive tasks [11]. This is a major reason why humans have succeeded in populating almost every place on this planet [12].

However, artificial intelligence (AI) is increasingly outperforming human intelligence when it comes to data processing speed; handling huge volumes of data; establishing connections between and within large sets of unrelated pieces of information, especially at first glance; very fast learning; and—as a result—forecasting and predicting the scenarios, behaviour, and functions of complex systems [13].

AI is already revolutionizing various science and technology sectors. It has tremendous impact on medicine [14], nutrition [15], diagnostics [16], environmental science [17], logistics [18], and robotics [19], and this impact is growing exponentially. Rational drug design, for example, is currently greatly enhanced by AI and holds promise for yielding more efficacious drugs in a shorter time [20]. In medical diagnostics, AI’s superb capability of pattern recognition is widely leveraged to improve and accelerate the early and accurate detection of disease states and deviations from healthy physiology, especially when combined with imaging technologies [21]. The fast-learning quality of AI has enabled robots to act autonomously and react to changing conditions and situations. In environmental research on climate change, for example, AI enables the forecasting of the behaviour of hugely complex systems, such as connected biospheres and atmospheres [22]. Logistic companies deploy AI to design and optimize networks of transportation and communication [18].

AI is therefore a key to improved health care, healthier food, a more sustainable food system, and—thereby—a healthier society and planet. AI has been described to potentially reduce global healthcare costs through more efficient and more directed discovery and development of nutritional interventions [23]. However, in nutrition, the power of AI is only beginning to be recognized and appreciated. AI can be deployed to discover bioactive, health-beneficial peptides in natural sources [6]. Living systems use peptides to communicate and regulate their functions. However, when these peptides reside in plant and food proteins, they are inactive unless they are unlocked from their parent proteins. Large peptide knowledge bases lay the foundation for predicting, localizing, unlocking, and testing peptides that deliver health benefits to humans and animals and make our food healthier and more sustainable [24]. In other words, AI can be used to better learn how to speak the language of nature and to teach the body to maintain or restore health and improve performance.

After the initial phase of the rather ad hoc discovery of natural bioactives, including peptides, high-throughput screening (HTS) has advanced both the speed and efficiency of the process [25]. It was and still is the attempt to leverage the biomolecular complexity and diversity of nature at large scale and with high automation. This said, the translation of these molecular discoveries into solutions for consumers and patients has been limited. Especially in nutrition, and in contrast to pharmaceutical applications, it was recognized that compound purity rarely correlates with compound bioactivity and bioefficacy: food bioactives typically exert multiple, individually subtle effects that—only when combined—may converge to a profound and lasting health benefit [26]. Hence, it is often the right blend of many bioactives and their optimal individual concentrations that make the difference. Furthermore, HTS represents a brute-force approach to fractionation and characterization of bioactives without much upfront guidance for possible translation and application, thereby resulting in a very high number of required bioactivity and bioefficacy tests, which are typically lower in throughput than the chemical screening is [27].

## 3. Artificial Intelligence for Prediction and Discovery of Bioactive Peptides

### 3.1. Concept and Computation

The key strategic advantage of using AI for the prediction and discovery of bioactive peptides is that purpose definition and design can be put in front of the entire process. This contrasts with traditional serendipitous or screening approaches. By putting AI in front of the peptide discovery and development process, the path from idea and concept to solution can be shortened from decades to a few years. The number of wet laboratory experiments and pre-clinical studies, which are time- and cost-determining factors, can be substantially reduced by upfront intelligent in silico design [6]. First, the desired benefit is defined, be it for human or animal health or for a healthier and more sustainable food solution; then, based on interrogable peptide knowledge repositories and machine learning, bioactive peptides can be predicted to exert such benefit; such predicted peptides can then be tested in vitro. The iterative process and integrated cycle of prediction and testing, which results in computational learning, can generate a feasible number of potent bioactive candidate peptides to then be validated in vivo, in a human, or in a food technology setting. Typically, three rounds of prediction and testing are performed to produce a list of lead peptides for further ingredient development. Consequently, AI can indeed guide and empower bioactive peptide discovery, food ingredient and dietary supplement design, manufacturing, and clinical validation [6,28]. Such AI-discovered, natural bioactive peptides were recently taken from concept to pre-clinically proven solutions [29,30,31,32].

### 3.2. Prediction of Peptide Properties

The target benefit is defined typically around the established or new value propositions of a private company or around an established or new research area of a public research institution. In the context of this manuscript this value proposition would be either in human health care or in the food/feed system’s sustainability. The molecular mechanisms underlying the specific target benefit(s), for example, consumer/patient benefits like physical mobility, immune balance, or metabolic control, can in many cases be identified through public and proprietary knowledge mining: both public-domain ‘flat files’ like publications or patents and publicly accessible databases can be downloaded and automatically mined for relevant information, e.g., by natural language processing [33]. Afterwards, manual—or rather cerebral—curation is needed to ensure the quality and relevance of the insourced information. This knowledge body can be combined with proprietary databases, which contain in-house generated information on peptide properties. Through interrogating these knowledge bases, the peptides involved in the identified key molecular mechanisms and the supposedly conferred target benefit(s) can be predicted by means of AI-powered mining, which relies on predictors for peptide functions and physicochemical properties [29,34].

**Figure 1:**
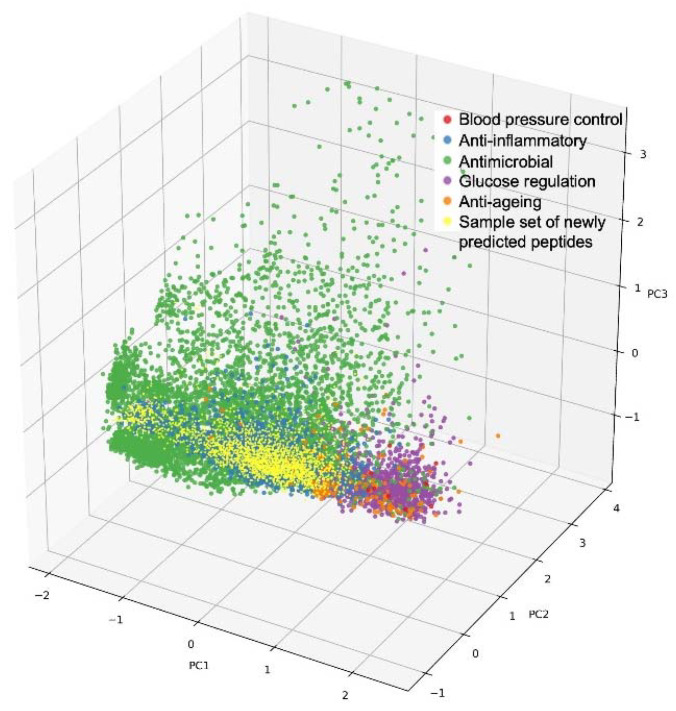
Illustration of a multi-dimensional peptide property space: 3D projection of annotated bioactive peptides according to three principal components (PC1-3), visualized as colored dots according to different health benefits. Red: blood pressure control; blue: anti-inflammatory; green: anti-microbial; magenta: glucose regulation; orange: anti-ageing; yellow: sample set of newly predicted peptides.

This can be further illustrated with the following analogy: every peptide has a unique sequence and a flexible three-dimensional (3D) structure. According to these 3D properties, every peptide can be projected into a 3D space containing all possible peptide 3D structures, thereby occupying a specific and unique position in that space. Now, the same can be envisaged with many more properties than just the 3D structure: peptides have numerous properties and various features that, when taken together, make up the entirety of individual peptide characteristics. According to these typically >100 characteristics, peptides can be projected into the high-dimensional space (representing these >100 characteristics), thereby again occupying one specific and unique position in that space. Visually speaking, this multi-dimensional space of peptides with their unique property combinations can then be “interrogated from the angle and with the depth of the desired property combination”, eventually “landing” on one or a few peptide positions that represent the best combination of the desired properties and hence the best hit(s) for a targeted, multi-faceted benefit [35]. This is illustrated in Figure 1.

The above-described peptide characteristics encompass the biological, biochemical, and physical properties, but they can also include peptide suitability and applicability resulting from these primary properties [24]. The biological, biochemical, and physical properties encompass amino acid sequence, molecular weight, hydrophobicity/hydrophilicity, basicity/acidity, solubility, and biochemical stability [36]. These features can determine the peptide’s suitability for specific applications. The key qualities for orally administered bioactive peptides are, for example, resistance to gastrointestinal digestion; transportability from the gut lumen to systemic blood circulation (active or passive transport across biological membranes); or the stability and half-life in human blood once absorbed from gut to blood [5]. The primary peptide properties may also determine the peptide’s relevance for commercial translation: the uniqueness of the peptide sequence influences the protectability of intellectual property; the amino acid sequence and length of a peptide impacts the manufacturing costs [37].

### 3.3. Natural Peptide Network (NPN) Design and Validation of Predicted Peptides and Designed Hydrolysates

Once a feasible and potent set of in vitro bioactive peptides has been identified and consolidated (from ‘hits to leads’), these candidate peptides can be blasted against all known plant and food genomes, proteomes, and peptidomes [36]. The purpose of this straightforward exercise is to identify the best natural source that holds the majority, if not all, of the desired peptide sequences in the plant and food parent proteins. When this optimal source is found—be it a grain, legume, vegetable, or any other edible plant, including not only terrestrial but also marine sources—the best protein hydrolysate with the resulting Natural Peptide Network (NPN), i.e., the combination of the predicted and initially validated peptide leads, needs to be designed [37]. Proteolytic digestion as such, be it in vivo (digestion) or in vitro (processing), may but does not necessarily produce bioactive peptides because these processes are not designed to do so. However, once a set of bioactive peptides deriving from plant and food proteins has been identified, the in vitro hydrolysis can be optimized to release the desired bioactive peptides, in addition to many others. This process represents a standard bioinformatic sequence analysis in the sense that the peptide locations in their parent proteins are determined and the adjacent potential cleavage sites are identified. With the latter in hand, the best proteases can be identified to be applied in combination to result in an optimized hydrolysis process and eventually yield the right combination and concentration of the targeted bioactive peptides [36]. In short, the best plant/food source in terms of the presence and abundance of the lead peptides in parent proteins is identified; based on the lead peptide sequence and its position within the parent protein in the plant/food source, the best enzymes for specific release of the lead peptides from their parent proteins are selected.

The predicted peptides with the best initial in vitro performance are then tested more extensively in relevant in vitro assays for bioactivity and toxicity. These peptides should be tested both individually and as the blend generated in the protein hydrolysate. Often, the hydrolysate exhibits greater bioactivity than the individual peptides, thanks to the combined, or even additive, effects of the multiple active principles present in the hydrolysate. These tests are typically performed over a range of peptide and hydrolysate concentrations, which can already to some extent inform on subsequent in vivo and in human studies. The advantage of bioactive food peptides is their inherent biosafety [8]. Therefore, after successful in vitro validation, the peptide and/or hydrolysate testing can often directly advance to a pilot pre-clinical study.

### 3.4. Natural Peptide Network (NPN) Manufacturing and Analysis

The optimal plant/food source is insourced, and the bulk protein material is extracted. The identified enzymes (proteases) are applied to a solution/suspension of this bulk protein material in different combinations and under different conditions (temperature, pH, time, concentration, etc.) [30,31]. The resulting protein hydrolysates are analyzed by MS-based peptidomics (see Section 4), which delivers a detailed peptide profile encompassing the NPN, i.e., the nearly complete hydrolysate peptidome [38]. Mass spectrometry enables the sequencing, identifying, and quantifying of peptides and proteins and is described in detail in the next section. Based on these preparations and analyses, the best procedure for manufacturing the desired protein hydrolysate with the optimal peptide profile is identified. The reproducibility of this optimized procedure is established in replicate manufacturing and analysis experiments. The optimized procedure is then established and used for further laboratory scale preparations, as well as for the pilot- and large-scale hydrolysate production for clinical trials and commercial purposes, respectively [32].

## 4. Mass Spectrometric Characterization of Bioactive Peptides

Mass spectrometry (MS) has developed into and is established as the most versatile and information-rich analysis method for biomolecules, especially for proteins [39] and peptides [38], but also for micro- and phyto-nutrients [40], metabolites [41], lipids [42], and DNA and RNA [43]. Mass spectrometry-based proteomics and peptidomics are the key platforms for the comprehensive analysis of proteins and peptides at the levels of identification, quantification, and structural characterization [44]. They have generated enormous data sets for protein- and peptide-related molecular biology, from molecular cell, tissue, and disease catalogues via complex biomolecular interaction networks, targets for drugs, and bioactives, to the structural and functional elucidation of complex biomolecules and even supra-molecular machineries [45].

Despite increasing efforts in top-down MS proteomics, e.g., the direct analysis of large, intact proteins for the fine characterization of antibodies [46] and the establishment of proteoform databases [47], MS proteomics, which is typically coupled online to upfront liquid chromatography (LC-MS), has been largely deployed as a bottom-up strategy [48]. That s, protein identification and quantification by sequencing of tryptic peptides representative of parent protein(s): the protein complement of a given sample (cell, tissue, body fluid, biopsy, extract, etc.) is digested by the protease trypsin, which generates “MS-friendly” peptides of typically 500 to 2500 Da with terminal arginine or lysine residues, which confer protonation sites in addition to inner-sequence basic side chains. The peptide mixture is subsequently separated via reversed-phase liquid chromatography (RP-LC) and directly and continuously infused into the mass spectrometer (MS) typically via electrospray ionization (ESI), where the eluting peptides are ionized (protonated), transferred into the gas phase, and then analyzed by intact mass (MS) and amino acid sequence (MS/MS), the latter two processes being executed in an alternating fashion “on the fly”. Peptide sequencing practically means the fragmentation of the peptide along its backbone and around the peptide bond and the generation of various peptide fragment types, which require the computational reassembly of the peptide sequence from those fragments [49].

This analysis process can be done either in data-dependent acquisition (DDA; the most intense intact peptides are selected and then sequenced) [50] or in data-independent acquisition mode (DIA; intact masses and sequencing are performed independently, without precursor ion selection, and the parent peptide-sequence context is reconstructed post hoc) [51]. Both acquisition methods have proven to be complementary. Finally, all sequenced and identified peptides are assigned to their parent proteins, which results in a proteome analysis of the entire sample.

The quantification of peptides and, thereby, proteins is done either by the label-free technique, i.e., the summation of spectral intensities across peptides and proteins (suited for less complex samples; limited by the facts that the MS response per se is analyte-dependent and that one LC-MS/MS run is required for each condition/sample type in question) [52], or by stable-isotope labelling methods, in which the protein or the tryptic peptide mixtures are derivatized with reagents carrying an isotopic signature, which is MS response-neutral, thereby allowing for the relative quantification of peptides and proteins between conditions blended in the same analytical sample and allowing for the multiplexed, simultaneous analysis of several conditions in one run [53]. Such stable-isotope labelling can be done in two different ways. When working with cells or bacteria, these living entities can be differentially grown with isotope-labelled media (e.g., with different stable-isotope coded amino acids) according to the conditions to be compared, which translates into a condition-specific signature to each such condition. This strategy is called ‘metabolic labelling’ and has been more specifically coined as the ‘stable-isotope labelling of amino acids in cell culture’ (SILAC) [54]. However, when working with samples derived from humans or other complex organisms, metabolic labelling is not an option. In those cases, the tryptic peptide mixtures are derivatized chemically by a set of reagents that are chemically identical (and therefore generate the same MS response when coupled to the same analyte) yet carry a specific isotope signature. As for SILAC, these differential signatures allow for relative quantification across several conditions within the same sample pool.

Most of the above-described proteomic features also apply to peptidomics, yet this latter discipline harbors specific conditions and challenges [55]. The main difference between proteomics and peptidomics is already indicated by their respective names: proteomics delivers the ‘proteome’, i.e., the totality of proteins, whereas peptidomics delivers the ‘peptidome’, i.e., the totality of peptides contained in a sample [56]. Such peptidomes can be enriched upfront by size-exclusion chromatography (SEC), by a membrane-based molecular-weight cut-off, by the precipitation of the proteins with acids or organic solvent [57] as such, and directly by LC-MS/MS. This peptide complement is typically much more heterogeneous than that of a tryptic peptide digest because the “native” peptides, i.e., those naturally contained in a sample, comprise a more diverse molecular space in terms of size, hydrophobicity, basicity (protonation efficiency), and fragmentation behaviour [58]. Moreover, a possibly bioactive ‘native’ peptide may occur in variants differing only in one or a few amino acids at the termini, and these ‘native’ peptide variants may be present individually in only very few copy numbers. This heterogeneity and complexity of a peptidome puts particular challenges on the LC-MS/MS analysis in terms of sensitivity and comprehensiveness [59].

Mass spectrometry, combined with tailored methods for peptide separation and enrichment, is the analytical platform of choice to identify, quantify, and characterize bioactive peptides as an analytical validation of AI prediction and discovery, and therefore it is an integral part of the development of bioactive peptide-based products [60]. MS-based peptidomics in this context is deployed for (a) characterizing bioactive peptides and the entire peptidome in the designed proteolytic hydrolysates of plant and food proteins [59]; (b) quantifying the desired bioactive peptides in vivo (that is, determining bioavailability in the gut lumen, blood, or target tissues; depending on the peptide’s place of action, the pre-clinical study design, and the sample availability) [61]; and (c) creating an experimental MS peptide library from plant and food proteins to analytically cover the technically accessible space of bioactive peptides [59]. In essence, artificial intelligence drives prediction and discovery, and mass spectrometry-based peptidomics provides analytical validation and proteolytically accessible peptidomes. These are complementary and synergistic platforms for the discovery and development of bioactive peptides as ingredients and drugs.

## 5. Conclusions

Bioactive peptides constitute a virtually unlimited reservoir of molecular solutions to human health care and a more sustainable food system. They co-evolved with mankind as biological messengers and regulators with specific functions. In particular, plant- and food-derived peptides represent a safe and sustainable complement of bioactives for nutritional and pharmaceutical applications. However, nature’s huge peptide reservoir has remained largely untapped to date because of inefficient discovery methods and the underestimated value of peptides as drugs and nutrients.

Artificial intelligence is changing this situation and unlocking this bottleneck. AI-driven bioactive peptide prediction and discovery, combined with downstream MS characterization, is a new development putting design and prediction up front, resulting in a benefit-directed, top-down approach as opposed to the traditional bottom-up, high-throughput screening. The establishment of large peptide knowledge bases and their AI-powered interrogation enables the efficient prediction of bioactive peptides for biofunctionalities within predefined health and sustainability benefits. The benefit-and-design-first principle and the computational zoom into most relevant bioactive candidates drastically reduces the number of in vitro and pre-clinical peptide tests and studies and thereby also reduces the development time and costs for peptide-based solutions. Artificial intelligence-guided and -driven bioactive peptide prediction, discovery, validation, and manufacturing will soon change the development of bioactives for better human and planetary health.

## Data Availability

Not applicable.
